# Supplementary material for: SpliceAI-10k calculator for the prediction of pseudoexonization, intron retention, and exon deletion
Source: Bioinformatics. 2023 Apr 6;39(4):btad179. doi: 10.1093/bioinformatics/btad179 (PMC10125908; doi:10.1093/bioinformatics/btad179)
Supplement: btad179_Supplementary_Data [file btad179_supplementary_data.zip › SAI10kcalc_supp_file1_final.pdf]

SUPPLEMENTARY FILE 1

SpliceAI-10k calculator for the prediction of pseudoexonization, intron retention, and exon deletion

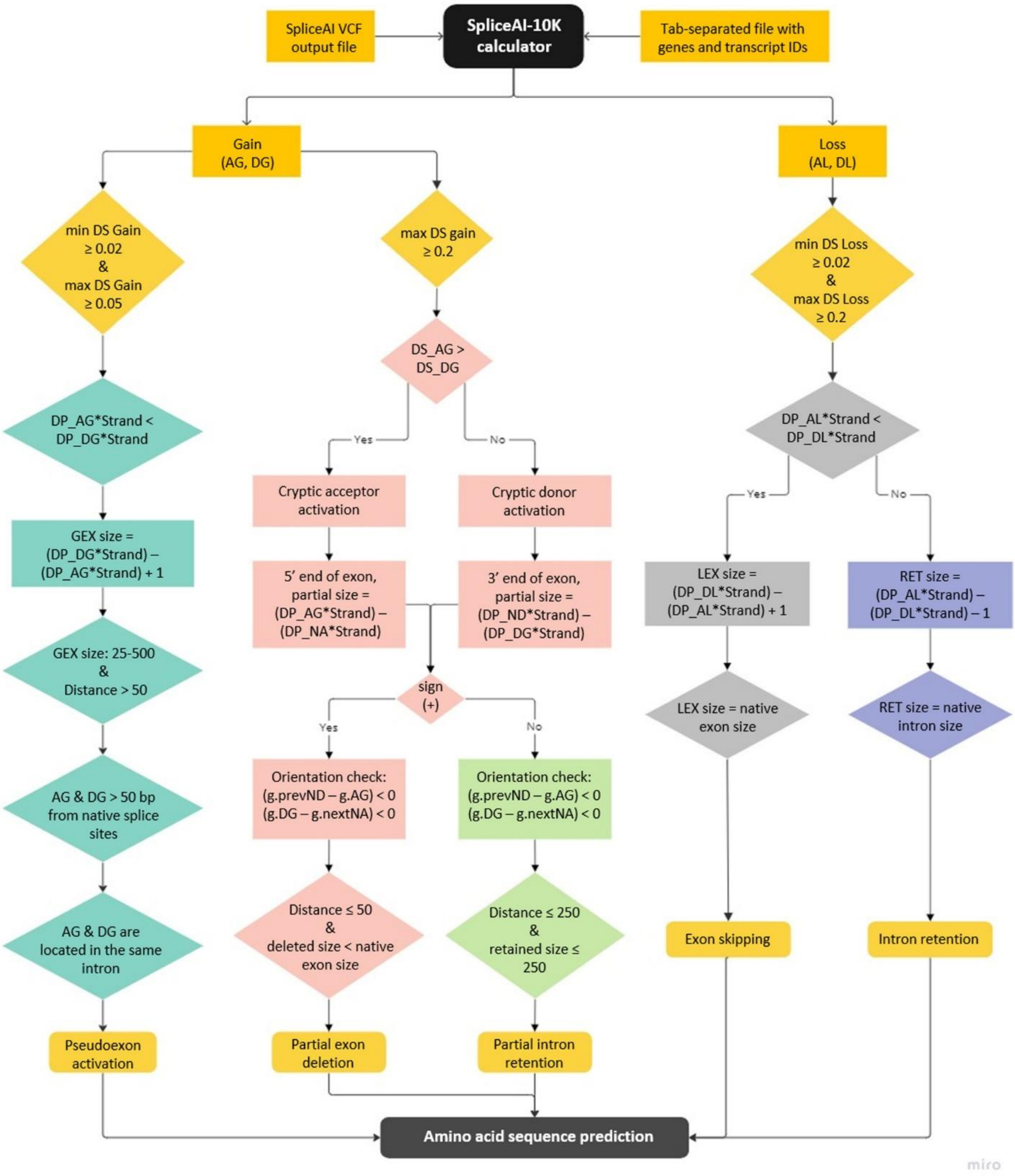

SpliceAI-10k calculator (R code version) decision flowchart. An intronic location threshold of >250 bp was set as an exclusive region for pseudoexonization. An intronic region within 50 bp of the exon-intron junction was set as a threshold for SNVs with potential to cause exon skipping. An intronic location within 250 bp of the exon-intron junction was set as the threshold for SNVs that lead to multiple aberration types (i.e. combinations of pseudoexonization, partial intron retention, and exon skipping). Abbreviations are spelled out below.

List of Abbreviations

DS_AG	delta score for acceptor gain
DS_AL	delta score for acceptor loss
DS_DG	delta score for donor gain
DS_DL	delta score for donor loss
DP_AG	delta position for acceptor gain
DP_AL	delta position for acceptor loss
DP_DG	delta position for donor gain
DP_DL	delta position for donor loss
DP_NA	delta position of the native acceptor in the given transcript
DP_ND	delta position of the native donor in the given transcript
Distance	variant intronic distance (bp) from the nearest exon-intron junction
g.AG	genomic location of acceptor gain
g.DG	genomic location of donor gain
g.prevND	genomic location of native donor upstream of gained acceptor
g.nextNA	genomic location of native acceptor downstream of gained donor
GEX	gained exon
LEX	lost exon
RET	retained intron
Strand	forward (1) or reverse (-1) strand orientation
VCF	variant call format

SPLICEAI-10K CALCULATOR OPTIONS

Gain, delta score threshold:
DS_AGDG_MIN (default ≥ 0.02)
DS_AGDG_MAX (default ≥ 0.05)

For pseudoexonization, one splice site must have a score that passes the maximum delta score threshold (DS_AGDG_MAX) for splice site gain, and its splice site pair must have a score greater than or equal to the lowest acceptable delta score (DS_AGDG_MIN).

Gained exon size range:
GEX_size_MIN (default ≥ 25)
GEX_size_MAX (default ≤ 500)

Gained exon size must be within the range bounded by the lower size limit (GEX_size_MIN) and upper size limit (GEX_size_MAX).

Loss, delta score threshold:
DS_ALDL_MIN (default ≥ 0.02)
DS_ALDL_MAX (default ≥ 0.2)

For exon skipping or whole intron retention, one splice site must have a score that passes the maximum delta score threshold (DS_ALDL_MAX) for splice site loss, and its splice site pair must have a score greater than or equal to the lowest acceptable delta score (DS_ALDL_MIN).

Cryptic splice site activation score threshold:
DS_AG (default ≥ 0.2)
DS_DG (default ≥ 0.2)

Created/activated cryptic acceptor or donor splice site must pass their respective delta score threshold (DS_AG or DS_DG).

The 0.2 upper threshold for exon skipping, whole intron retention, and cryptic splice site activation is based on the lower limit set by the developers of SpliceAI (Jaganathan et al., 2019). For deep intronic variants, the 0.05 upper threshold for pseudoexon gain is supported by the findings of Moles-Fernández et al. (2021). The 25–500 bp exon size range encompasses the optimal size for efficient splicing that is between 50–250 bp (Movassat, Forouzmand, Reese, & Hertel, 2019) and expected to capture most gained pseudoexons.

More information can be found at https://github.com/adavi4/SAI-10k-calc.

Supplementary References

Jaganathan, K., Kyriazopoulou Panagiotopoulou, S., McRae, J. F., Darbandi, S. F., Knowles, D., Li, Y. I., Kosmicki, J. A., Arbelaez, J., Cui, W., Schwartz, G. B., Chow, E. D., Kanterakis, E., Gao, H., Kia, A., Batzoglou, S., Sanders, S. J., & Farh, K. K.-H. (2019). Predicting Splicing from Primary Sequence with Deep Learning. *Cell, 176*(3), 535-548.e524. doi:10.1016/j.cell.2018.12.015

Moles-Fernández, A., Domènech-Vivó, J., Tenés, A., Balmaña, J., Diez, O., & Gutiérrez-Enríquez, S. (2021). Role of Splicing Regulatory Elements and In Silico Tools Usage in the Identification of Deep Intronic Splicing Variants in Hereditary Breast/Ovarian Cancer Genes. *Cancers, 13*(13), 3341. doi:10.3390/cancers13133341

Movassat, M., Forouzmand, E., Reese, F., & Hertel, K. J. (2019). Exon size and sequence conservation improves identification of splice-altering nucleotides. *RNA, 25*(12), 1793-1805. doi:10.1261/rna.070987.119
